# Immunotherapy by Immune Checkpoint Inhibitors and Nuclear Medicine Imaging: Current and Future Applications

**DOI:** 10.3390/cancers12020371

**Published:** 2020-02-06

**Authors:** Pierre Decazes, Pierre Bohn

**Affiliations:** 1 Department of Nuclear Medicine, Henri Becquerel Cancer Center, 76000 Rouen, France; pierre.bohn@chb.unicancer.fr; 2 LITIS-QuantIF-EA (Equipe d’Accueil) 4108, IRIB, Faculty of Medicine, University of Rouen, 76000 Rouen, France

**Keywords:** positron emission tomography, programmed cell death 1 receptor, diagnostic imaging, CTLA-4 Antigen, Immunotherapy, Adoptive, radioactive tracers, radionuclide imaging, CD8-Positive T-Lymphocytes

## Abstract

Immunotherapy by using immune checkpoint inhibitors is a revolutionary development in oncology. Medical imaging is also impacted by this new therapy, particularly nuclear medicine imaging (also called radionuclide imaging), which uses radioactive tracers to visualize metabolic functions. Our aim was to review the current applications of nuclear medicine imaging in immunotherapy, along with their limitations, and the perspectives offered by this imaging modality. *Method*: Articles describing the use of radionuclide imaging in immunotherapy were researched using PubMed by April 2019 and analyzed. *Results*: More than 5000 articles were analyzed, and nearly 100 of them were retained. Radionuclide imaging, notably ^18^F-FDG PET/CT, already has a major role in many cancers for pre-therapeutic and therapeutic evaluation, diagnoses of adverse effects, called immune-related adverse events (IrAE), and end-of-treatment evaluations. However, these current applications can be hindered by immunotherapy, notably due to atypical response patterns such as pseudoprogression, which is defined as an increase in the size of lesions, or the visualization of new lesions, followed by a response, and hyperprogression, which is an accelerated tumor growth rate after starting treatment. To overcome these difficulties, new opportunities are offered, particularly therapeutic evaluation criteria adapted to immunotherapy and immuno-PET allowing us to predict responses to immunotherapy. Moreover, some new technological solutions are also promising, such as radiomic analyses and body composition on associated anatomical images. However, more research has to be done, notably for the diagnosis of hyperprogression and pseudoprogression. *Conclusion*: Immunotherapy, by its major impact on cancer and by the new patterns generated on images, is revolutionary in the field of medical images. Nuclear medicine imaging is already established and will be able to help meet new challenges through its plasticity.

## 1. Introduction

Cancers are a proliferation of abnormal cells that can have the ability to disrupt the host’s adaptive immune response to avoid a control by tumoricidal attack [1]. This process can be overcome by immunotherapy, which aims to stimulate the body’s immune system against cancer cells.

Immunotherapy may be broken down into three main families. Firstly, passive cancer immunotherapy, which consists of the use of antibodies directed against tumor proteins, or adoptive T-cell therapy targeting a tumor receptor; secondly, active immunotherapy involving cytokines and vaccines; thirdly, immunomodulatory therapy including immune checkpoint inhibitors (ICI) and targeting, more specifically, the tumor microenvironment. 

Immunotherapy using ICI—notably anti-CTLA-4 (cytotoxic T-lymphocyte antigen 4), anti-PD-1 (programmed cell death protein-1), and anti-PDL-1 (PD1 ligand) antibodies—is a recent successful therapeutic approach that reactivates the immune system against cancers [2,3]. In a meta-analysis combining 19 studies involving 11,640 patients treated by ICI or other drugs, a team observed that a durable response (i.e., a progression-free survival exceeding three times the median progression-free survival of the whole population) occurred for 25% of the patients treated by ICI, which is a far better rate when compared to other drug classes (11%) [4]. However, as the therapeutic approach of ICI is different from usual cytotoxic approaches—notably by generating inflammations rather than direct lysis—medical imaging has to be interpreted differently than when using cytotoxic chemotherapy.

Thus, ICI introduces new challenges in medical imaging. Pre-therapeutic examinations have to identify prognostic factors—linked, for example, to the extent and the burden of the disease—as well as predictive factors of the response to immunotherapy, since it is known that not all patients will benefit from immunotherapy. New imaging techniques, called immunoimaging, target tumor or inflammatory cell and are promising for this indication. Concerning therapeutic evaluation, follow-up examinations must allow the detection of non-responders; in order to change the therapeutic line, this detection has to be done early, particularly in cases of hyperprogression—which is an acceleration of tumor growth rate sometimes observed after starting immunotherapy [5,6]. At the same time, to avoid the premature discontinuation of treatment beneficial to the patient, a real disease progression should not be confused with a pseudoprogression, which is an increase in the size of lesions, or the visualisation of new lesions, followed by a response [6,7]. Finally, end-of-treatment examinations should allow patients to safely stop immunotherapy in the event of durable response [6].

These challenges concern morphological and anatomical imaging, including computed tomography (CT), magnetic resonance imaging (MRI), and ultrasound. However, they also concern functional imaging, particularly nuclear medicine imaging.

To produce images, nuclear medicine uses radiotracers, which are a combination of a radioactive atom and a tracer targeting a metabolic function. These radionuclide images can be produced by detecting in vivo radiotracers with, in case of gamma (γ) radioactivity, a gamma camera (used notably to create a 3D single photon emission computed tomography, alias SPECT), or with, in case of a positron (β+) radioactivity, positron emission tomography (PET), which is usually coupled with a CT to form a PET/CT.

As morphological modifications, functional modifications due to ICI are visible on nuclear medicine images and must be known not only by nuclear physicians but also by oncologists requesting examinations. In addition, nuclear medicine, by its high lability and sensitivity, can offer many solutions, now and in the future, for the new challenges raised by ICI.

The objective of this review is to conduct a systematic review of the articles concerning nuclear medicine and ICI, with a summary of the pathophysiology and a focus on changes already made in the current clinical routine and those to come.

## 2. Materials and Methods 

A literature review was performed using Medline (PubMed). The research was conducted on 2 April 2019 to identify articles published between 2008 and 2019 with a search key combining date of publication and MeSH terms linked to immunotherapy, nuclear medicine and medical imaging.

The PubMed key search was: (“2008” [Date—Create]: “2020” [Date—Create]) AND (“programmed cell death 1 receptor” [MeSH Terms] OR “CTLA-4 Antigen” [MeSH Terms] OR “CD8-Positive T-Lymphocytes” [MeSH Terms] OR “Immunotherapy, Adoptive” [MeSH Terms] OR “CTLA-4 Antigen” [nm] OR “PDCD1 protein, human” [nm] OR nivolumab [nm] OR “pembrolizumab” [nm] OR “durvalumab” [nm] OR “ipilimumab” [nm] OR “avelumab” [nm] OR “atezolizumab” [nm] OR “immunotherapy” [All Fields] OR “PD-L1” [All Fields] OR “PD-1” [All Fields] OR “CTLA-4” [All Fields]) AND (“radioimmunotherapy” [MeSH Terms] OR “Alpha Particles” [MeSH Terms] OR “Beta Particles” [MeSH Terms] OR “radionuclide imaging” [MeSH Terms] OR “Diagnostic Imaging” [MeSH Terms] OR “radioactive tracers” [MeSH Terms] OR “imaging” [All Fields] OR “PET” [All Fields] OR “radiology” [All Fields] OR “nuclear medicine” [All Fields] or radioactiv* [All Fields])

**Figure 1:**
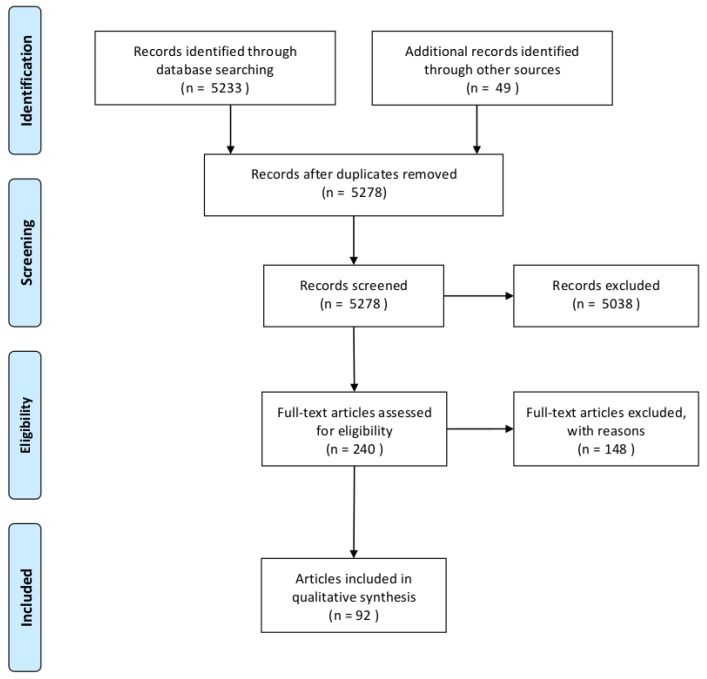
Preferred Reporting Items for Systematic Reviews and Meta-Analyses (PRISMA) flowchart for studies selection.

Search results were judged for relevance using the title, abstract, and full text for inclusion in the analysis. Two researchers (one nuclear medicine physician and one radiopharmacist) performed the research and the critical analysis of the articles. A Preferred Reporting Items for Systematic Reviews and Meta-Analyses (PRISMA) flowchart [8] of the studies selection process is shown in Figure 1.

## 3. Physiopathology

^18^F-FDG, exploring glucose metabolism, is the main PET radiotracer used in nuclear medicine imaging. However, ^18^F-FDG is not specific to tumor cells and also targets immune cells. Since immunotherapy works by inducing an inflammatory response, it is therefore difficult to differentiate ^18^F-FDG uptakes related to tumor cells from those due to inflammation.

**Figure 2:**
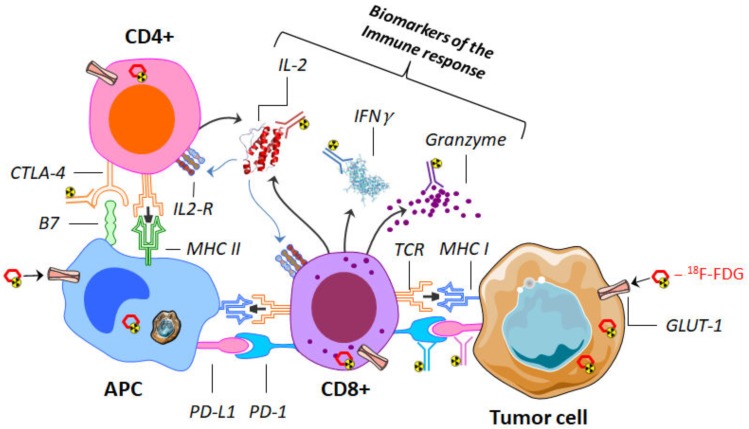
Representation of the interaction between CD4 and CD8 lymphocytes with an antigen-presenting cell (APC) (here a macrophage) and a tumor cell. Different targets can be imaged by use of several labeled antibodies (immuno-PET). These targets may be immune-checkpoints (such as cytotoxic T-lymphocyte antigen 4 (CTLA-4), PD-L1, and its receptor programmed cell death protein-1 (PD-1)) or biomarkers of the immune response (such as interferon γ (IFNγ), granzyme and interleukin-2 (IL2)). ^18^F-fluorodeoxyglucose (18F-FDG), on the other hand, makes it possible to assess the expression of the glucose transporter (GLUT-1); it can be incorporated indifferently, both in tumor and immune cells. PET: positron emission tomography.

To overcome this limitation, an understanding of the mechanisms of ICI is helpful in identifying potential new targets for nuclear medicine imaging. The physiopathology is summarized below and shown in Figure 2.

Cell-mediated immunity is performed by immune cells independently from circulating antibodies. These cells are macrophages and natural killer cells from the innate system or T cells including helper, regulatory, and killer T cells from the adaptive immune system. Among them, cytotoxic T cells expressing CD8 as a co-receptor of T-cell receptor (TCR) cause the cell death of tumor cells after releasing serine proteases such as granzymes. However, the cytotoxic T cell activity is regulated and modulated by others cells such as myeloid-derived suppressor cells (MDSC) and regulatory T cells expressing CD4. 

The cytotoxic T cell allows an immune response against tumor cells. The specificity of the immune response is driven by the interaction between cells expressing major histocompatibility complex receptor (MHC) and TCR. Briefly, major histocompatibility complex class I (MHC-I), displaying an antigen from tumor cells, will be recognized by the TCR of a cytotoxic T cell. However, co-inhibitory and co-stimulatory signals regulate this immune response. Co-stimulatory signals such as interleukin-2 (IL-2) or interferon γ (IFNγ) improve the immune response against foreign antigens. On the contrary, the co-inhibitory signals alleviate the immune response to allow self-tolerance. Thus, PD-L1 and PD-L2 expressed on the membrane surface of tumor cells are recognized by PD-1 of the cytotoxic T cell, and this leads to stopping the immune response. Moreover, if CTLA-4 expressed by regulatory T cells binds to B7 expressed by antigen-presenting cells (APCs), the immune response declines [9].

The signals shared between tumor cells, T cells, macrophages, and dendritic cells in the tumor microenvironment, due to the interaction of their ligand–receptor pairs, are known as immune checkpoints. The tumor cells that release and/or express mediators of immune suppression, such as PD-L1 and PD-L2, have a chance to proliferate by inhibiting the immune response. 

The pharmacology of ICIs is based on the reactivation of the immune response against tumors rather than a direct effect on tumor cells [10,11]. The ICIs are only monoclonal antibodies targeting and blocking the co-inhibitory signals of either programmed cell death protein 1 (PD-1) or cytotoxic T-lymphocyte antigen-4 (CTLA-4)—which are cytotoxic T cell surface receptors—or a ligand of PD-1 (PD-L1) from tumor cells or antigen-presenting cells [12]. These molecules are used in the oncologic field and harness the immune system to help to fight tumor cells [10].

## 4. Medical Imaging: Baseline Examination

If the response to ICIs seems better compared to other drug classes [4], predictive biomarkers to determine which patients will respond to immune checkpoint inhibitors are useful in making the right treatment choice [13]. Medical imaging—particularly nuclear medicine—has an essential role to play in this field because it offers, in one examination, the possibility of characterizing tumors that are often multifocal and cannot therefore be all biopsied.

Compared to other medical imaging modalities such as CT or MRI, nuclear medicine imaging generally provides the advantage of a highly sensitive whole-body functional examination, the specificity depending on the radiotracer used. The specificity can be low for a multifunctional radiotracer (such as ^18^F-FDG, whose uptakes concern tumor and inflammatory cells) to very high for radiotracers targeting specifically an immune cell receptor or an active inflammatory signal (however, such radiotracers are still in development). Concerning the disadvantages, the spatial resolution of nuclear medicine imaging is worse than CT and MRI, and radiation protection must be taken into account. Many radiotracers are currently studied with a summary (potential interest, name, target, development phase) presented in Table 1.

It has to be noticed that the radionuclide used to label the radiotracer has to be chosen carefully. In particular, it must be able to chemically bind to the tracer molecule either by covalent or by coordinate bonds. The emitted radioactivity must also be adapted to the imaging method used with the gamma (γ) ray emitter (Technetium ^99m^Tc, Indium ^111^In), for the gamma camera and positron (β+) emitter (Fluorine ^18^F, Copper ^64^Cu, Gallium ^68^Ga, Zirconium ^89^Zr) for PET—the spatial resolution of the PET being inherently better than that of the gamma camera. Moreover, the radioactive half-life of the radionuclide has to be adapted to the physiological process being explored: for short processes (a few hours), ^99m^Tc, ^18^F, and ^68^Ga can be chosen, but for long processes (a few days) such as those observed with antibodies, ^111^In, ^64^Cu, and ^89^Zr with longer radioactive periods have to be used. Finally, the deposited energy dose, which varies according to the radionuclides and the tracers’ clearance, must be taken into account to ensure that it is as low as possible.

### 4.1. ^18^F-FDG PET/CT

^18^F-fluorodeoxyglucose (^18^F-FDG) is a multifunctional PET/CT radiotracer exploring glucose metabolism—including tumor metabolism and inflammation—and is a well-established nuclear medicine examination performed to explore cancer, notably for the extension assessment [14].

Beyond the extension assessment, baseline ^18^F-FDG total metabolic tumor volume (TMTV), measured by segmenting all tumors on PET images, could be an interesting parameter, as it can be easily (semi)-automatically determined, and as it presents an important prognostic value in many cancers and treatments, including immunotherapy. Therefore, Ito et al. have shown for 142 patients with melanoma treated by ipilimumab that the median overall survival (OS) was significantly lower for patients with high TMTV (10.8 months 95% CI 5.9–15.8 months) than with low TMTV (26.0 months 95% CI 3.0–49.2 months) [15]. In a multivariate analysis including TMTV and significant clinical parameters (including age and active brain metastases), TMVT was still a significant prognostic factor [15]. Similar results have been observed for non-small cell lung carcinoma (NSCLC) treated by ICI [16].

Beyond its role in the initial extension assessment, the ^18^F-FDG PET could also be useful in determining the PD-1/PD-L1 status, on which the response to immunotherapy depends. Effectively, PD-L1 promotes glycolytic metabolism in tumor cells, while this glucose consumption by tumors metabolically restricts T cells, notably by dampening their glycolytic capacity [17]. As a result, PD-L1 protein expression was significantly correlated to glucose transporter 1 (GLUT1) expression, which is the transporter of ^18^F-FDG, in lung adenocarcinoma [18,19] and squamous-cell carcinoma [20]. In a meta-analysis about lung cancer including three studies (718 patients), ^18^F-FDG PET SUVmax (maximal standardized uptake value reflecting tumor activity) and PD-L1 expression were slightly correlated (Spearman’s correlation 0.36 (95% CI: 0.22; 0.50)), although this low value does not allow the SUVmax to be used as a replacement for the PDL1 status in lung cancer [21]. For bladder cancer, a study including 63 patients has shown too that SUVmax was significantly higher in PD-1/PD-L1-positive patients but also that PD-1 and PD-L1 status could be predicted using a SUVmax cut-off value of 22.7, with quite good accuracies of 71.4% and 77.8%, respectively [22]. It has to be added that ^18^F-FDG SUV is not only significantly correlated to PD-1/PD-L1 status, but also to other biomarkers of interest when immune-checkpoint inhibitors are considered, including CD8 tumor infiltration by lymphocytes (TILs) [23].

^18^F-FDG PET/CT could also be used to determine indirect predictive factors for response to immunotherapy treatment. Therefore, this examination could reflect the gut microbiota, whose analysis could be favorable to predict the treatment effect of immunotherapy, as gut microbiota seems linked to the therapeutic response [24]. Thus, in a retrospective pilot study involving 14 patients with metastatic melanoma treated with ipilimumab in the first line, a team observed that colonic, pre-therapeutic ^18^F-FDG PET SUVmax seems lower for responding patients with mean SUVmax equal to 1.33 ± 0.04, 2.2 ± 0.46, and 3.33 ± 2.67 for individuals with complete response, partial response, and progressive disease, respectively [25]. This could be explained by an association between low bacterial load and higher physiologic colonic ^18^F-FDG uptake due to a shift in colonic metabolism from short-chain fatty acids, produced by colonic bacteria, to glycolysis when the bacterial load is low [26]. However, these promising preliminary results have to be confirmed.

Another indirect predictive factor is the determination of body composition parameters on the anatomical CT associated with the PET. Therefore, in a study including 55 patients with NSCLC treated by nivolumab, whole-body subcutaneous fat mass measured automatically on the CT of the PET was a significant prognostic factor with a better prognosis for fatty patients (HR = 0.75, p = 0.006 in multivariate analysis) [27,28], possibly because adipocytes in the human obese subcutaneous fat mass release several pro-inflammatory cytokines and chemokines, which contribute to the establishment and maintenance of local and systemic inflammation [29].

Nevertheless, ^18^F-FDG is not as tumor-specific as we might wish, notably for response assessment during immunotherapy. One of the main problems with ICIs is related to the inflammatory reactions that recall neutrophils and macrophages and activate T cells on the tumor site. This antineoplastic activation determines the high consumption of FDG by immune cells and, consequently, a loss of specificity for this radiotracer.

### 4.2. Immunoimaging

Despite the remarkable success of ICIs in clinical practice, the efficacy and responsiveness of these agents vary greatly among different tumor types and across individuals [30]. Biomarkers determined on tumor biopsy, including PD-1 and PD-L1 measured with immunohistochemistry (IHC), can help to select patients. However, these biomarkers are limited, as some patients can have a response without them, while others can have no response with them [31]. A possible explanation is that a single biopsy may not capture the heterogeneity across various tumor lesions in a patient or within a single lesion [31]. Therefore, the use of radiotracers to predict responses to immunotherapy is becoming increasingly important to better select patients, as they have the possibility to characterize the whole tumors in a single non-invasive examination. Among the possible methods, “immuno-PET”, combining antibodies (or antibody fragments) with PET radionuclide (positron emitter), takes advantage of the specificity and affinity of antibodies and the sensitivity of PET [32].

For immuno-PET, targets can be general T cell markers (such as CD3, CD4, and CD8), immune checkpoints (such as PD-1, PD-L1, or CTLA-4) or biomarkers of the immune response (such as interferon-γ, interleukin-2, and granzyme B) [33,34]. The absence of impact on the functionality of in vivo T cells also needs to be considered [35]. Many radiotracers have been evaluated in preclinical models, with a large “immunoimaging toolbox” already available [36], but few of them have ever been tested in clinical trials [33,36,37]. A summary of the usable radiotracers and target is presented in Figure 2 and in Table 1. 

Among the radiotracers tested in clinical trials, antibodies targeting checkpoints inhibitors, notably PD-1/PD-L1, have shown promising results. In a study including 22 patients with three tumor types (bladder cancer, NSCLC, or breast cancer) with pre-therapeutic PET/CT with ^89^Zirconium-labeled atezolizumab (anti-PD-L1), PET uptakes were heterogeneous and generally high in tumors [38]. Uptake was also high in lymphoid tissues and at sites of inflammation [38]. Interestingly, progression-free survival was better correlated with the pretreatment PET signal than with IHC-based predictive biomarkers, with, for example, a hazard ratio equal to 11.7 (95% CI: 3.3–62.7) for PET versus 2.6 (95% CI: 0.8–13.6) for an immunohistochemistry analysis of PD-L1 [38]. These results for the survival are possibly linked to the heterogeneity of the PD-L1 expression across different tumor localizations. Comparable results were obtained in another study evaluating two anti-PD-1/PD-L1 radiotracers (a ^18^Fluor-labeled anti-PD-L1 Adnectin and an anti-PD-1, ^89^Zirconium-labeled nivolumab) on 13 patients with advanced NSCLC [39]. Other PD-L1-specific radiolabeled peptides exist. Notably, the so-called “[^64^Cu]WL12”, which is a 14-amino acid circular peptide “WL12”, with a binding interface on PD-L1 overlapping with PD-1 and PD-L1 therapeutics, radiolabeled with ^64^Cu, a positron (β+) emitter [40]. A preclinical study on mice has shown that this radiotracer could generate highly contrasted images within 120 minutes after the injection [41], which is a usual time period for conducting nuclear medicine examinations. Moreover, this peptide could measure the PD-L1 occupancy and the pharmacokinetics of PD-L1 immune-checkpoint inhibitor non-invasively and independently of the type of antibody chosen for treatment [40].

Imaging of the biomarkers of immune response could also be interesting. Interferon-γ (IFNγ) immuno-PET (^89^Zr-anti-IFN-γ) offers the possibility to explore the activated lymphocytes inside tumors, including CD8+ cytotoxic T lymphocytes [42]. In a preclinical study, the tumor uptake with this radiotracer seemed more specific than CD3 immuno-PET (^89^Zr-anti-CD3), which targets the general T-cell population localized in the tumor but also in other lymphoid tissues, and it could help to discern activated versus anergic/dysfunctional status [42]. Another interesting target of immune response could be the protease granzyme B (GZP), which is secreted by cytotoxic CD8+ and has a key role for immune-induced apoptosis [43]. In preclinical tumor models, a highly specific peptide PET imaging agent for GZP was predictive of the response to immune-checkpoint inhibitors, with high signal tumors responding to therapy and with 93% sensitivity and 94% negative predictive value [44]. Clinical studies have yet to be done.

Finally, a radiotracer for a gamma camera using monophotonic gamma radioactivity, which is different from the positron (β+) radioactivity used for PET, is also possible. A study on 16 patients with NSCLC demonstrates that a ^99m^Tc-labeled anti-PD-L1-single domain antibody was feasible in humans safely and with acceptable dosimetry [45]. The image acquisition was also possible rather quickly after the injection at 2 h [45]. However, if the ratio between the tumor uptake and blood pool at 2 h was significantly correlated to the PD-L1 immunohistochemistry (ρ = 0.68, *p* = 0.014) and the uptake ratio was lower in tumors with ≤1% PD-L1 expression (1.89 versus 2.49, *p* = 0.048), the results seem too limited to be used in clinical practice [45]. Another radiotracer for a gamma camera is ^99m^Tc-interleukin-2 (^99m^Tc-IL2), which could be used to detect TILs and distinguish between true progression and pseudoprogression [46]. A pilot study has demonstrated the safety and feasibility of ^99m^Tc-IL2 imaging, but further studies are needed [46].

### 4.3. Radiomics and Complex Quantitative Parameters

Radiomics is a set of methods used to take advantage of medical imaging and extract quantitative features that can characterize the tumor phenotype. A very large number of features can be extracted (manually or automatically) from medical images (e.g., CT, MRI, PET) and then correlated to tumor characteristics and clinical outcomes using machine learning algorithms.

In a retrospective study using four independent cohorts of patients, Sun et al. have shown the usefulness of this method in determining the tumor infiltration by CD8 cells on contrast-enhanced CT images and with a signature combining eight features [47]. Despite the relatively low area under the curve of the score for this prediction (AUC = 0.67; 95% CI 0.57–0.77), the signature was able to predict an objective response to anti-PD-1 and PD-L1 therapy, notably at 3 months (*p* = 0.049), as well as overall survival in univariate (median overall survival was 24.3 months in the high radiomic score group versus 11.5 months in the low radiomic score group; *p* = 0.0081) and multivariate analyses [47]. Another study explored the interest of radiomics as a non-invasive biomarker for responses to cancer immunotherapy on 1055 primary and metastatic lesions from 203 contrast-enhanced CTs from patients with advanced melanoma and NSCLC, undergoing anti-PD1 therapy [48]. They found on a lesion-based approach, reflecting the metastatic condition, that lesions with heterogeneous density and more compact and spherical (high volume/surface ratio) were associated with a better response [48]. Concerning ^18^F-FDG PET radiomic analysis and PD-1/PD-L1 expression, a team found in 53 oropharyngeal or hypopharyngeal cancer patients that several PET-derived textural features, describing the organization of tumor pixels, can provide information to determine tumor PD-L1 expression in head and neck carcinoma [49]. However, no clear and validated textural model distinguishing high and low PD-L1 expression is described, and more studies have yet to be done. Finally, delta-radiomics (∆-radiomics), studying changes in radiomic features (e.g., texture within the nodule) on serial images could be useful to assess the effectiveness of therapy as well as predict early treatment response, but this domain as yet to be explored [50].

Complex quantitative parameters could be also interesting, such as compartmental parameters describing the kinetics of radiotracers more precisely than SUV. However, such parameters have not shown an added value compared to SUV in a population of 25 patients with metastatic melanoma treated with immunotherapy [51]; therefore, further studies are still needed.

## 5. Therapeutic Evaluation

**Figure 3:**
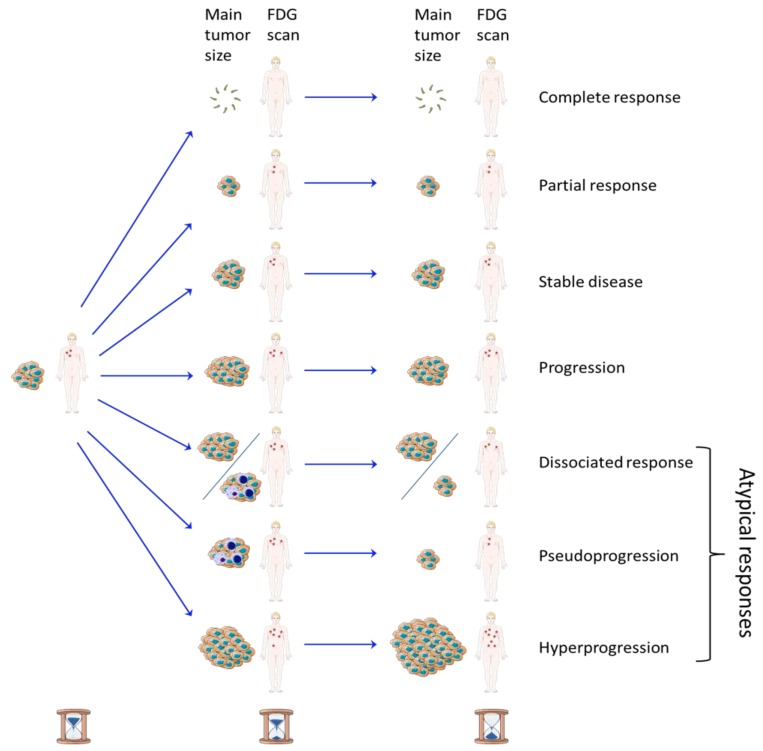
Patterns of response according to the size and number of the tumors and in function of time of examination. Complete response, partial response, stable disease, and progression are the four classical patterns of response in oncology. Concerning the atypical responses sometimes observed with immune-checkpoint inhibitors, a dissociated response corresponds to lesions shrinking and others growing; pseudoprogression is an initial increase in tumor size and/or number due to inflammation followed by a decrease, and hyperprogression is an accelerated tumor growth rate after starting treating.

^18^F-FDG PET/CT is a routinely used for the examination of therapeutic evaluation of cancers. However, ICIs, treating cancer by inducing inflammation, question its interpretation and the time of execution, because this radiotracer presents an uptake in the case of active cancer but also of inflammation. Comparable challenges are observed for anatomical images. Different patterns of response according to the time of examination are shown in Figure 3.

### 5.1. Standard Therapeutic Assessment Scales

The therapeutic evaluation of cytotoxic chemotherapy in morphological imaging is based on the fact that increasing lesion size and/or the appearance of new lesions after treatment indicates progression and therapeutic failure [12]. Many therapeutic evaluation criteria exist for morphological imaging, including Response Evaluation Criteria in Solid Tumors (RECIST) 1.1, which uses unidimensional single-diameter measurements [52]. Compared to morphological imaging, such as CT, functional imaging including ^18^F-FDG PET/CT can provide an earlier response assessment. One of the situations where therapeutic assessment by functional imaging by ^18^F-FDG PET may be much earlier than morphological imaging is gastrointestinal stromal tumors (GISTs) treated by imatinib. While measuring anatomical responses through morphological imaging (CT) often requires many months, 18F-FDG PET can predict responses within one day to one week [53]. Some therapeutic evaluation criteria exist in nuclear medicine, including the PET Response Criteria in Solid Tumors (PERCIST) 1.0 for solid tumors [54] and the Lugano classification for lymphomas [55].

### 5.2. Limitations due to Atypical Tumor Response Patterns

The therapeutic scales used to evaluate cytotoxic chemotherapies are generally based on a classification with four patterns of response: complete response, partial response, stable disease, or progression. However, “atypical” tumor response patterns that do not belong to these four categories can be observed with ICIs, which makes the therapeutic evaluation of these treatments a real challenge.

Pseudoprogression is an atypical tumor response pattern defined as an increase in the size of lesions or the visualization of new lesions, followed by a response [6,7]. This effect could be explained by the initial T-cell tumor infiltration, with an apparent increase in the tumor burden disconnected to the tumor cell proliferation [56]. In studies, rates of pseudoprogression never exceeded 10%; therefore, they stayed rare compared to effective disease progression [6].

Dissociated responses, observed when some lesions shrink and others grow, are another atypical tumor response of ICIs. One study reported dissociated responses in 7.5% of NSCLC patients treated with anti-PD1/PD-L1 agents with a better survival observed than true progressions [57]. Another study with 50 patients having NSCLC treated by ICI and followed by ^18^F-FDG PET showed that dissociated response occurred in 26% (five patients) of the 19 patients whose treatment was continued after initial progression with a clinical benefit for these patients [58]. 

Nevertheless, due to these atypical responses, classical criteria, such as RECIST or PERCIST, can be limited to the therapeutic assessment of immune checkpoint inhibitors; some diseases can be classified as “progressive”, while this is the response to the treatment that occurs [56,58].

### 5.3. Updated Therapeutic Assessment Scales for Immunotherapy

To avoid a misdiagnosis in therapeutic evaluation between progressive disease and inflammation, a modification of the scales has been proposed. In anatomical and morphological imaging, at least four modified criteria have already been proposed: irRC (immune-related response criteria) (2009) [56], irRECIST (immune-related RECIST) (2013) [59], iRECIST (immune RECIST) (2017) [7], and imRECIST (immune-modified RECIST) (2018) [60]. Some differences exist between these scales, but they have all in common the need to confirm a progressive disease on a new examination, which is usually at 4 weeks [61]. The interest of these updated scales has been highlighted in a study including 160 patients with NSCLC with follow up performed by computed tomography. Patients with atypical responses, according to the iRECIST and irRECIST, represented 11% of the RECIST 1.1 so-called progressive patients, and they had superior overall survival compared to patients with the true disease progression [57].

If the increase in the size or appearance of new lesions due to pseudoprogression can mislead the therapeutic evaluation in radiological imaging, the ^18^F-FDG PET metabolic evaluation can also be misleading. Effectively, ^18^F-FDG is not a tumor-specific radiotracer, and the inflammatory response, which displaces neutrophils, macrophages, and activated T cells to the tumor site, also leads to the uptake of ^18^F-FDG [62].

Therefore, an effort has been made in nuclear medicine with modified scales proposed, including, for solid tumors, PECRIT (PET/CT Criteria for the early prediction of Response to Immune checkpoint inhibitor Therapy) (2017) [63], PERCIMT (PET Response Evaluation Criteria for Immunotherapy) (2018) [64], imPERCIST5 (immunotherapy-modified PERCIST up to five lesions) (2019) [65], and iPERCIST (immune PERCIST) (2019) [66]. Table 2 resumes the proposed criteria for therapeutic evaluation by ^18^F-FDG PET of solid tumors treated by immune checkpoint inhibitor.

**Figure 4:**
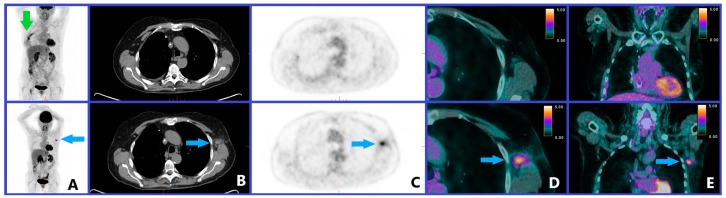
Clinical case of a patient with a previous history of treated left breast cancer and melanoma of the right leg with melanoma pulmonary metastasis recently operated. The images of the upper line show the PET/CT examination before the start of immune checkpoint inhibitor (pembrolizumab), and the images in the lower line show the PET/CT examination performed 9 months after the start of the treatment with (in A) a maximum intensity projection (MIP) PET image; in B, an axial CT slice; in C, an axial PET slice; in D, an axial PET/CT fused slice; in E, a frontal PET/CT fused slice. The green arrow on the pre-therapeutic PET MIP indicates the post-operative inflammation of melanoma pulmonary metastasis. The blue arrows on the follow-up PET/CT examination indicate that an intense left axillary lymph node uptake (SUVmax 4.7) appeared, which was considered suspicious of disease progression when it was found to be an inflammatory reactive lymph node in histological analysis.

However, these nuclear medicine criteria are quite different, and therefore, standardization is needed [67]. It should be noticed that only iPERCIST introduces the need to confirm a progressive disease such as the updated radiological criteria [66]; however, such a control may limit the risk of a false positive PET scan. A dissociated response is also not considered in these scales, while it seems important as the patients experiencing these response patterns seems to have a clinical benefit of immunotherapy [58]. Moreover, the populations used to define these criteria were relatively small (from 20 patients for PERCRIT [63] to 60 patients for imPERCIST [65]), and studies with more patients (and, therefore, more cases of atypical tumor responses) are needed to validate these criteria. The localization of the uptake should also be considered, as an immune activation induced by checkpoint treatment can be observed in tumor-draining lymph nodes [68] that can induce an ^18^F-FDG uptake and be misinterpreted as progression, as seen in Figure 4. 

Different scales for solid tumors and hematological tumors have to be considered as the response patterns appear to be different. For lymphomas, modified Lugano criteria have been proposed with the establishment of lymphoma response to immunomodulatory therapy criteria (LYRIC) [69]. Comparable to the category "unconfirmed progressive disease" used for solid tumors by iRECIST and iPERCIST [7,66], LYRIC has introduced the category “indeterminate response” (IR) when an increase in tumor burden, new lesions, and/or increased ^18^F-FDG uptake is observed with subsequent imaging within 12 weeks, which is recommended to confirm the true disease progression versus pseudoprogression [69]. However, concerning Hodgkin lymphoma, the contribution of LYRIC compared to the standard Lugano classification seems limited, because pseudoprogressions seem very rare in this disease. Thus, no pseudoprogression was reported in the two studies, including a total of 60 patients with refractory or relapsed Hodgkin lymphoma treated with immunotherapy and having an early therapeutic evaluation (2 to 3 months) by ^18^F-FDG PET/CT [70,71]. Moreover, in a retrospective multicentric study including 45 patients with Hodgkin lymphoma treated by nivolumab, the classification at the ^18^F-FDG PET performed at 2.0 months (interquartile range: 1.7–3.7 months) was identical between Lugano criteria and LYRIC, since all 16 progression events on this examination classified as an indeterminate response per LYRIC were confirmed as progressive diseases on subsequent evaluations [72]. In most other types of lymphoma, including follicular lymphoma and diffuse large B cell lymphoma, ICIs have been rarely used to date, because they are less effective, and therefore, there is a lack of data regarding their therapeutic evaluation [73].

### 5.4. Hyperprogression Disease and Early Therapeutic Response Evaluation

Hyperprogression disease is an acceleration of the tumor growth rate (TGR) with a ΔTGR (variation of TGR per month) exceeding 50% at the first evaluation compared to pretreatment kinetics [74]. This phenomenon is quite common for patients treated with ICI, as it was observed in 7% (12 of 189) patients with solid tumors [5] and is associated with high metastatic burden and poor prognosis [74].

As hyperprogression disease drives toward early death, notably when it occurs in the first 6 weeks of treatment, the anticipated first radiological evaluation during PD-1/PD-L1 inhibitor treatment, including ^18^F-FDG PET/CT, has to be discussed in order to identify them [74]. 

^18^F-FDG PET/CT performed early after the initiation of immunotherapy could not only detect hyperprogression but also participate in the therapeutic evaluation, notably by detecting early responders and non-responders more easily than with CT. Therefore, in a study with 24 patients with NSCLC treated by nivolumab, metabolic responses determined by ^18^F–FDG PET at 1 month (especially total lesion glycolysis, TLG, which is the product of the TMTV and the mean SUV) were closely associated with therapeutic response and survival, while it was difficult to distinguish between responders and non-responders on morphological changes on CT scans [75]. In another pilot prospective study of 10 patients with unresectable metastasized melanoma, the responding patients could be reliably identified as early as two weeks after the start of the therapy [76], which was consistent with the usual early metabolic response, compared to the generally later anatomical response.

Interim evaluation, performed after the first two cycles of immunotherapy, is also possible with interesting results, as was shown in a population of 41 patients with unresectable metastatic melanoma treated by ipilimumab. The PERCIMT classification showed a sensitivity of 93.6%, a specificity of 70.0%, and an accuracy of 87.8% to predict clinical benefit—including stable disease, partial response, and complete response (31 patients)—and those showing no clinical benefit including progressive disease (10 patients) according to the best clinical response of patients, which was assessed at a median of 21 months [77]. 

### 5.5. Abscopal Effect

Radiation therapy can induce the death of cancer cells and can activate the immune system [78]. The abscopal effect is a tumor regression observed in metastases distant from the local treatment site, such as the primary irradiation site [78]. The combination of radiotherapy and immunotherapy can have the advantage of controlling disease locally with potential systemic and lasting immune effects [78]. PET/CT can observe such responses, even if, until now, only case reports have been reported [79,80,81]. 

## 6. Diagnosis of Side Effects in Nuclear Medicine Imaging

**Figure 5:**
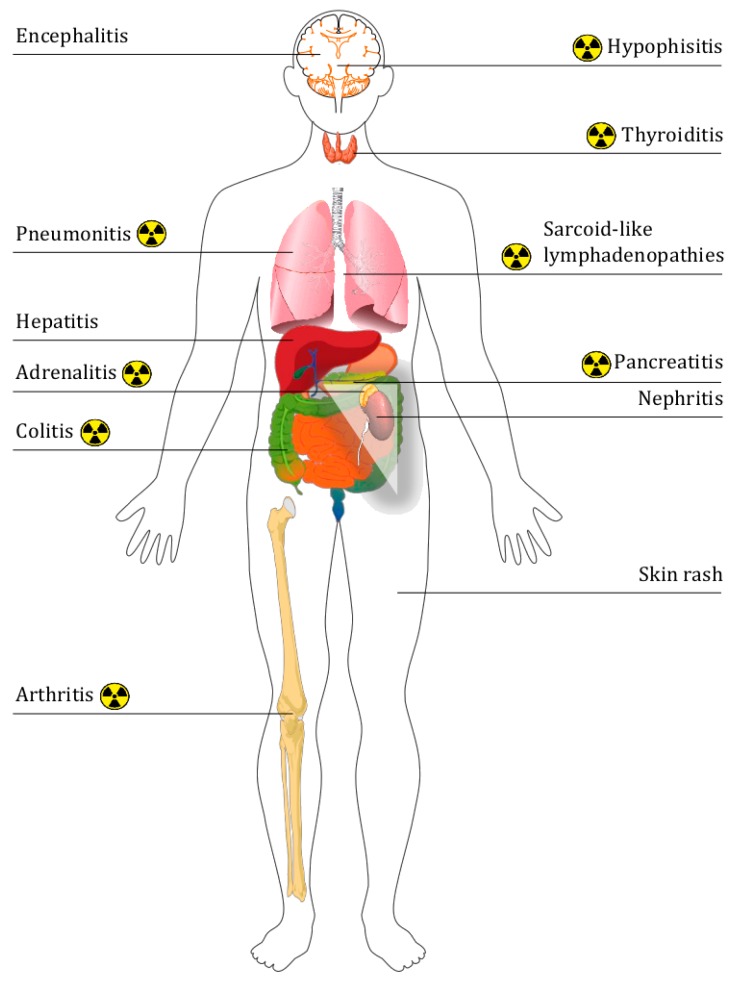
Summary diagram of the different immune-related adverse events. The nuclear symbol represents the side effects that can be visualized by ^18^F-FDG PET/CT.

The physiological objective of CTLA4 and PD-1/PD-L1 is the blockade of auto immune responses. When these pathways are inhibited, T-cell inflammatory responses can be triggered to target cancer; however, they also target some healthy tissues. The attacks on healthy tissue can generate immune-related adverse events (IrAE), the scope of which is large [82] and the gravity of which, though generally low, can be severe (around 10% patients with anti-PD-1/PD-L1 experienced severe IrAEs [83]) and even potentially life threatening if not diagnosed and treated. Therefore, the permanent discontinuation of immune checkpoint inhibitors is advised in patients with high-grade ocular, hepatic, pancreatic and/or pulmonary IrAEs [82]. IrAEs are generally detected by clinicians, but medical imaging can help to find early signs of unknown IrAEs, with radiologically evident IrAEs reported in up to approximately 30% of patients [84]. Moreover, to avoid errors in therapeutic evaluation, these IrAEs have to be differentiated from active cancerous disease, notably from metastatic progression. Figure 5 resumes the discoverability by ^18^F-FDG PET/CT of different IrAEs.

In a monocentric retrospective study with the objective of describing the IrAE characteristics on medical imaging and their detection rate, Mekki et al. found 39 (74%) abnormal medical imaging findings on 53 patients who had a co-occurrence of irAEs and medical imaging (CT, PET/CT, MRI, US of radiography) within 15 days [85]. Among the 12 performed ^18^F-FDG PET/CT, 10 (83%) were abnormal with thoracic sarcoid-like reaction, enterocolitis, thyroiditis, hypophysitis, and pancreatitis [85].

Regardless of their severity, the occurrence of an IrAE could also be linked to the therapeutic response by revealing the immune response. In a study of 41 patients with metastatic melanoma treated by anti-CTLA-4 (ipilimumab), four patients among the 31 having disease control had a sarcoid-like mediastino-hilar lymphadenopathy diagnosed on the follow-up ^18^FDG PET/CT, while this side effect was not observed on the 10 patients having progressive disease [86]. The same team performed a study on 16 patients with BRAF-mutation positive metastatic melanoma treated by a combination of vemurafenib and ipilimumab with longitudinal ^18^F-FDG PET/CT for the follow-up. Seven patients developed imaging signs on PET/CT of at least one immune-related adverse event, with colitis and arthritis being the most frequent ones (five and four events, respectively), and these patients had a significantly longer progression-free survival than those without irAEs (*p* = 0.036) [87]. Another team found concordant results on a retrospective study of 40 patients with three types of cancer (malignant melanoma, malignant lymphoma, and renal cell carcinoma) treated by ICIs and followed by ^18^F-FDG PET/CT. They found that a PET-detectable immune-related adverse event indicated a favorable outcome (nine of 11 patients with IrAE had complete response at final evaluation) with in particular thyroiditis, which was seen earlier than other IRAE and could provide an early indicator of the efficacy of immunotherapy [88]. Therefore, immune-related inflammation has to be reported even if they are not necessarily associated with clinically significant IrAE [89]. Illustrations of ^18^F-FDG PET-detectable immune-related adverse effects are presented in several articles [85,88,90].

Another early sign of immune activity is an inversion of the liver-to-spleen ratio (normally >1) [89], possibly by reflecting the immune activation preceding T cell proliferation [91], but also the reactive nodes in the drainage basin of the primary tumor [89], which could be wrongly diagnosed as cancerous lymph nodes (cf Figure 4).

Finally, if immune checkpoint inhibitors do not promote infections, immune-related adverse effects often require immunosuppressive treatment, which in turn increases the risk of developing serious infections [92], and such infections can be observed on imaging.

## 7. End-of-Treatment Assessment

^18^F-FDG PET imaging may also help better predict long-term outcomes compared to standard computed tomography (CT) response criteria. Therefore, a team has shown in a retrospective study of 104 patients with melanoma, who received anti-PD-1 as monotherapy (67%) or combined with ipilimumab (31%), that while only a small proportion of patients had a complete response at 1 year on CT (28%), most patients with a partial response on CT have a complete metabolic response on PET (68% of the 66% having a partial response) [93]. Moreover, almost all patients with complete metabolic response on PET at 1 year had ongoing response to therapy thereafter (78% had discontinued treatment and 96% had ongoing response) [93]. ^18^F-FDG PET could help to decide on the continuation/discontinuation of therapy [93], notably when durable response is observed [6].

## 8. Conclusions

In this systematic review, we have observed that immune checkpoint inhibitors have a major impact on nuclear medicine imaging with changes in its interpretation, including the consideration of induced inflammation by new therapeutic evaluation criteria.

New perspectives are also emerging, with a central role for nuclear medicine being the prediction of the response to immune checkpoint inhibitors, notably by the use of new radiotracers, such as immuno-PET, or new analysis techniques, including radiomics and body composition analyses.

## Abbreviation

^18^F-fluorodeoxyglucose^18^F-FDGAntigen presenting cellsAPCComplete Metabolic ResponseCMRComplete responseCRComputed tomographyCTCytotoxic T cellsCTLsCytotoxic T-lymphocyte antigen 4CTLA-4Gastrointestinal stromal tumorsGISTGlucose transporter 1GLUT1Interferon γIFNγInterleukine-2IL-2Immune checkpoint inhibitorICIImmune-modified RECISTimRECISTImmune PERCISTiPERCISTImmune RECISTiRECISTImmune-related adverse effectIrAEImmune-related RECISTirRECISTImmune-related response criteriairRCImmunotherapy-modified PERCIST up to 5 lesionsimPERCIST5Non-small cell lung cancerNSCLCMajor histocompatibility complexMHCMagnetic resonance imagingMRIMyeloid-derived suppressor cellsMDSCPartial Metabolic ResponsePMRPartial ResponsePRPET/CT Criteria for early prediction of Response to Immune checkpoint inhibitor TherapyPECRITPET Response Criteria in Solid TumorsPERCISTPET Response Evaluation Criteria for ImmunotherapyPERCIMTProgrammed cell death protein-1PD-1Programmed cell death protein-1 ligandPDL-1Progression diseasePDPositron emission tomographyPETPreferred Reporting Items for Systematic Reviews and Meta-AnalysesPRISMAResponse Evaluation Criteria in Solid TumorsRECISTSingle photon emission computed tomographySPECTStable diseaseSDStandardized uptake valueSUVStandardized uptake value normalized par lean body massSULT-cell receptorTCRTotal lesion glycolysisTLGTotal metabolic tumor volumeTMTVTumor-infiltrating lymphocytesTILs

## Figures and Tables

**Table 1:** Radiotracers studied in the context of immunotherapy with potential interest, radiotracers, targets and development phase. GLUT-1: glucose transporter 1, NSCLC: non-small cell lung carcinoma.

Potential Interest	Radiotracers	Targets	Development Phase
Imaging of tumor cells and inflammation	18F-FDG	GLUT-1 and hexokinase	Market Authorization
Imaging of checkpoints inhibitors	89Zr-Atezolizumab	PD-L1	Phase I (Lymphoma, Breast cancer, Renal cell carcinoma)
	18F-Adnectin	PD-L1	Preclinical (Rodent)
	18F-PD-L1 ([18F]BMS-986192)	PD-L1	Phase I/II (Melanoma, NSCLC, Oral cancer)
	99mTc-anti-PD-L1(99m-Tc-NM-01)	PD-L1	Phase I (NSCLC)
	64Cu-WL12	PD-L1	Preclinical (Rodent)
	89Zr-Nivolumab([89Zr]-BMS-936558)	PD-1	Preclinical (Primate)
	89Zr-Ipilimumab	CTLA-4	Phase II (Metastatic melanoma)
Imaging of biomarkers of immune response	89Zr-IFNγ	*IFN* *γ*	Preclinical (Rodent)
	68Ga-NOTA-GZP	Granzyme	Preclinical (Rodent)
	18F-IL2 ([18F]FB-IL2)	IL2	Open label (metastatic melanoma); Phase I (renal transplant rejection)
	99mTc-IL2	IL2	Phase I (metastatic melanoma)

**Table 2:** Proposed criteria for therapeutic evaluation by ^18^F-FDG PET/CT of solid tumors treated by immune checkpoint inhibitor. RECIST: Response Evaluation Criteria in Solid Tumors, PERCIST: PET Response Criteria in Solid Tumors, PECRIT: PET/CT Criteria for the early prediction of Response to Immune checkpoint inhibitor Therapy, PERCIMT: PET Response Evaluation Criteria for Immunotherapy; imPERCIST5: immunotherapy-modified PERCIST up to five lesions, iPERCIST: immune PERCIST, SUL: standardized uptake value normalized by lean body mass.

Criteria for therapeutic evaluation	RECIST 1.1	PERCIST 1.0	PECRIT	PERCIMT	imPERCIST5	iPERCIST
Year	2009	2009	2017	2018	2019	2019
References	[52]	[54]	[63]	[64]	[65]	[66]
Population study	Not defined	Literature review	Retrospective analysis of 20 advanced melanoma patients treated with anti-CTLA-4 (*n* = 16) or anti-PD-1/PDL-1 (*n* = 4)	Retrospective analysis of 41 metastatic melanoma patients treated with ipilimumab	Retrospective analysis of 60 metastatic melanoma patients treated with ipilimumab	Retrospective analysis of 28 NSCLC patients treated with nivolumab
Objective	Assessment of treatment outcomes	Starting point for clinical trials and structured reporting	Predict clinical benefit	Predict clinical benefit	Determine prognosis of patients	Identify patients who can benefit most from treatment
Modality	CT, MRI (^18^F-FDG PET complementary modality)	Functional imaging (^18^F-FDG PET)	Combination of anatomic (CT) and functional imaging (^18^F-FDG PET)	Functional imaging combined with CT (^18^F-FDG PET/CT)	Functional imaging (^18^F-FDG PET)	Functional imaging (^18^F-FDG PET)
Time	Undetermined	Undetermined	Early: 3–4 weeks after beginning immunotherapy	3 months after beginning immunotherapy	3 months after beginning immunotherapy	2 months after beginning immunotherapy
Measurable lesions	≥ 10 mm, 5 lesions in total, maximum 2 per organ	Minimum tumor SUL 1.5 times the mean SUL of the liver, up to five target lesions per patient	RECIST 1.1 PERCIST 1.0	Functional size (measured on fused PET/CT) > 1.0 or 1.5 cm Up to 5 target lesions per patient	PERCIST 1.0 (up to five lesions)	PERCIST 1.0
New lesion	As progressive disease	As progressive disease	RECIST 1.1 (As progressive disease)	As progressive disease based on number and functional size (see PMD below)	Do not define PMD but are included in the sum of SULpeak. PMD if summed SULpeak variation is >30%	As progressive disease (but must be confirmed if still unconfirmed PMD)
Complete Metabolic Response (CMR)	Disappearence of all lesions	Disappearance of all metabolically active tumors	RECIST 1.1 (Disappearence of all lesions)	Complete resolution of all preexisting ^18^F-FDG-avid lesions; no new ^18^F-FDG-avid lesions	Disappearance of all metabolically active tumors	Complete resolution of ^18^F-FDG uptake within the target lesion
Partial Metabolic Response (PMR)	≥30% decrease from baseline	Reduction in SULpeak in target lesions by a at least 30%, and absolute drop in SUL by at least 0.8 SUL units.	RECIST 1.1 (decrease in target lesion diameter sum ≥ 30%)	Complete resolution of some preexisting ^18^F-FDG-avid lesions, no new ^18^F-FDG-avid lesions	Reduction in SULpeak in target lesions by ≥30% and absolute drop in SUL by ≥0.8 SUL units	≥30% decrease in the target ^18^F-FDG SULpeak
Stable Metabolic Disease (SMD)	Neither progressive disease (PD), partial response (PR) PR, nor complete response (CR)	Neither PMD, PMR, nor CMR	1) RECIST 1.1 (Neither PD, PR or CR) 2) Evaluation of change in SULpeak of the hottest lesion of: - >15.5% (clinical benefit predicted) - ≤15.5% (no clinical benefit predicted)	Neither PMD, PMR, nor CMR	Neither PMD, PMR, nor CMR	Neither PMD, PMR, nor CMR
Progressive Metabolic Disease (PMD)	≥20% increase in the nadir of the sum of target lesions, with a minimum of 5 mm	Increase in SULpeak of > 30% or the appearance of a new metabolically active lesion	RECIST 1.1 (≥20% increase in the nadir of the sum of target lesions, with a minimum of 5 mm)	Four or more new lesions of < 1 cm in functional diameter or Three or more new lesions of > 1 cm in functional diameter or Two or more new lesions of more than 1.5 cm in functional diameter	>30% increase in SUL peak, with >0.8 SUL unit increase in tumor SULpeak, from baseline scan in a pattern typical of tumor and not of infection/treatment effect.	≥30% increase in SULpeak or advent of new ^18^F-FDG-avid lesions: unconfirmed PMD (UPMD)
Confirmation PMD	Not applicable	Not applicable	Not applicable	Not applicable	Not applicable	Need to be confirmed by a second PET at 4–8 weeks later: confirmed PMD (CPMD)
